# Behavioral Investigation of the Influence of Social Categorization on Empathy for Pain: A Minimal Group Paradigm Study

**DOI:** 10.3389/fpsyg.2012.00389

**Published:** 2012-10-17

**Authors:** Benoît Montalan, Thierry Lelard, Olivier Godefroy, Harold Mouras

**Affiliations:** ^1^Laboratoire de Neurosciences Fonctionnelles et Pathologies, UFR de Médecine, Université de Picardie Jules VerneAmiens, France; ^2^Laboratoire de Psychologie et Neurosciences: Intégration Cognitive du Neurone à la Société, Université de RouenMont-Saint-Aignan, France; ^3^Structure Fédérative de Recherche en Santé CAP-Santé, Université de Picardie Jules Verne, Amiens and Université de Reims-Champagne-ArdennesReims, France; ^4^Service de Neurologie, Center Hospitalier Universitaire d’AmiensAmiens, France

**Keywords:** empathy for pain, ingroup bias, social categorization, minimal group paradigm

## Abstract

Research on empathy for pain has provided evidence of an empathic bias toward racial ingroup members. In this study, we used for the first time the “minimal group paradigm” in which participants were assigned to artificial groups and required to perform pain judgments of pictures of hands and feet in painful or non-painful situations from self, ingroup, and outgroup perspectives. Findings showed that the mere categorization of people into two distinct arbitrary social groups appears to be sufficient to elicit an ingroup bias in empathy for pain.

## Introduction

Empathy is thought to play a critical role in social interactions in motivating prosocial behavior (e.g., Dovidio et al., 1990). This human ability is a psychological construct regulated by both cognitive and affective components (e.g., Decety and Jackson, 2004; Shamay-Tsoory et al., 2009). The affective component involves sharing the emotional experiences of others, while the cognitive component involves thinking about and understanding the mental states of others.

Research on the psychological and neural mechanisms of empathy has substantially grown this past decade, focusing more specifically on how we share the pain of others, one of the most basic and universal human experiences. In this regard, it has been recently demonstrated that response to other’s pain depends on the social relationships between the observer and the individuals experiencing the outcome. For example, affective links (e.g., Singer et al., 2006), perceived similarities (e.g., Perry et al., 2010), and social memberships (e.g., Xu et al., 2009) are likely to modulate the level of empathy experienced by the observer toward agent’s pain. More precisely, number of studies on empathy for pain in intergroup contexts has provided evidence of an empathic bias toward racial ingroup members (Xu et al., 2009; Avenanti et al., 2010; see Chiao and Mathur, 2010, for a review).

In the field of social psychology, it is well-known that people are remarkably adept at dividing up the social world into *us* versus *them*, and that this propensity has important affective, cognitive, and behavioral consequences such as prejudice, stereotype, and discrimination. These various implications are particularly induced when categorical information such as race, gender, or age, provide visually salient cues to group membership (Fiske and Neuberg, 1990). However, studies based on the Minimal Group Paradigm (MGP; Tajfel et al., 1971) have demonstrated that the mere categorization of individuals into two social groups on the basis of arbitrary criteria, such as whether they tend to overestimate or underestimate the number of dots on a screen (Diehl, 1990), is sufficient to produce similar consequences as compared to natural groups. Now, no studies used the MGP, in research on empathy for pain. Thus, the present study examined whether the mere categorization of people into distinct arbitrary social groups is sufficient to elicit an ingroup bias in empathy for pain. To this end, participants were shown pictures of people in painful or non-painful situations and were instructed to imagine themselves or imagine members of two minimal groups (ingroup vs. outgroup) in the same situations. Participants then had to rate the level of perceived pain according to the different perspectives (e.g., Jackson et al., 2006).

## Materials and Methods

### Participants

Thirty-six native French speakers were included (eight males; mean age = 21.5 ± 2.2). None had prior or current treatment for any psychiatric disorder or neurological condition (Godefroy et al., 2010). The study conformed to the IASP’s guidelines and was approved by the local ethics committee (CPP Nord Ouest 2, Amiens, France). All participants provided informed written consent.

### Procedure

Upon arrival in the lab, participants provided informed consent and were asked to fill out the Interpersonal Reactivity Index (IRI; Davis, 1983; French version: Guttman and Laporte, 2002). The IRI is a multidimensional measure composed of 28 self-report items using a 5-point Likert scale and designed to evaluate different dimensions of empathy. The subscales of the IRI consist of four subscales of seven items. The *Fantasy* (*F*) subscale assesses the tendency to imaginatively transpose oneself into fictional situations (e.g., *I really get involved with the feelings of the characters in a novel*). The *Perspective Taking* (PT) subscale assesses the tendency to spontaneously adopt the psychological point of view of others (e.g., *I try to look at everybody’s side of a disagreement before I make a decision*). The *Empathic Concern* (EC) subscale assesses the tendency to experience feelings of sympathy and concern for others in distress (e.g., *I often have tender, concerned feelings for people less fortunate than me*). The *Personal Distress* (PD) subscale assesses the tendency to experience distress and discomfort in response to distress in others (e.g., *In emergency situations, I feel apprehensive, and ill-at-ease*). In the present study, and as in previous ones (e.g., Rankin et al., 2006), we chose to focus on two IRI subscales only, which measure the cognitive (PT subscale) and affective (EC subscale) components of empathy.

After filling out the IRI, participants were informed that the session was part of a larger project investigating the relationship between cognition and emotion. They were comfortably seated at a viewing distance of 70 cm from a computer monitor and instructed to perform a *cognitive task* in order to determine their *cognitive profile*. A dot-estimation task was used to divide subjects into two fictive groups: 18 participants were categorized as *underestimators*, i.e., those who allegedly had underestimated the number of dots in a series of stimulus displays, while 18 others were categorized as *overestimators*, i.e., those who allegedly had overestimated the number of dots in a series of stimulus displays. Finally, the experimenter gave additional information to the participants about the supposed differences between the two groups in order to reinforce their feeling of membership (see also Ashburn-Nardo et al., 2001; Pinter and Greenwald, 2011) and, more specifically, to avoid potential effects of asymmetry in terms of status or power (e.g., Sachdev and Bourhis, 1987; e.g., “Previous research has shown that people who overestimate the number of dots tend to process perceptual information in a bottom-up fashion. That is, you tend to examine the finer details of new stimuli, and then form an overall impression. In contrast, people who underestimate the number of dots tend to process perceptual information in a top-down fashion. That is, they tend to form an overall impression, and then examine the finer details of new stimuli. However, none of these two modes of processing is better than the other one.”).

Just after the minimal categorization procedure, participants performed an *emotion task*, i.e., a pain judgment task in which they were shown 36 static visual stimuli, consisting of 18 color pictures showing hands and feet in painful situations and 18 color pictures of hands and feet in non-painful situations (Jackson et al., 2005). Adobe Photoshop^®^ (Adobe Systems, Inc.) was used to resize the images to approximately 500 × 375 pixels (screen resolution: 1024 × 768 pixels). The stimuli were presented in three blocks (themselves, members of the ingroup, and member of the outgroup) of 36 trials each, for a total of 108 trials. At the beginning of each block, the participants were instructed to imagine themselves, a member of the ingroup, or a member of the outgroup in the displayed situations, and to rate the level of perceived pain according to the different perspectives. The order of the blocks was counterbalanced on a between-subjects basis. The trial sequence started with a fixation cross for 500 ms. Then the stimulus was presented until participants responded. Immediately after the onset of the stimulus, subjects were instructed to deliver their ratings by pressing with the right hand one of nine computer keys (with scores ranging from 0 = *no pain* to 8 = *very severe pain*). After responses, an inter-stimulus interval (ISI) of 1000 ms was added.

Following the pain judgment task, subjects completed a 20-item adjective rating scale, including 10 positive items and 10 negative items (Montalan et al., 2011), to measure ingroup bias. The negative stimuli had a mean valence rating of −3.04 ± 1.47, while the positive stimuli had a mean valence rating of 3.85 ± 0.93. Participants rated how descriptive each item was for each target group. Responses range from 0 = *does not describe* to 8 = *describes completely*. The negative adjectives of the scale were reverse scored. Thus, an evaluative score (ES) used to measure ingroup bias was calculated by subtracting for each participant the outgroup evaluation score from the ingroup evaluation score (outgroup bias: ES < 0, no bias: ES = 0, and ingroup bias: ES > 0).

### Statistical analyses

For the pain judgment task, pain ratings and response times were submitted to two repeated-measure analyses of variance (ANOVA) with Stimulus (Pain vs. No-pain) and Perspective (Self vs. Ingroup vs. Outgroup) as within-subject factors. For the ingroup bias measure, difference of evaluation scores from zero was evaluated with a one-sample *t*-test. Finally, Pearson’s correlation coefficients between the subjects’ performance in the pain judgment task, the evaluation scores and the IRI scores (PT and EC scores) were also calculated. For all statistical analyses, the α was fixed at 5%.

## Results

### Pain judgment task

#### Pain ratings

**Figure 1:**
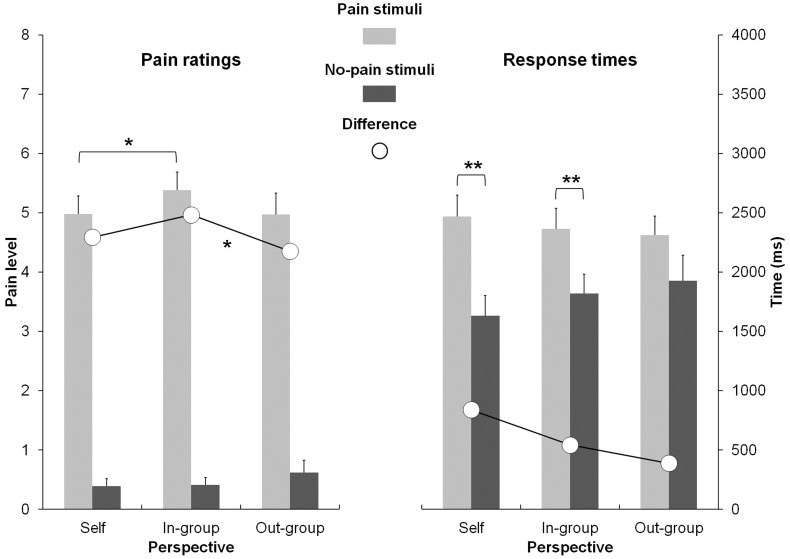
**Pain ratings (left) and response times (right) as a function of stimuli (pain vs. no-pain) and perspective (self vs. ingroup vs. outgroup)**. *<0.05; **<0.001.

The two-factor ANOVA revealed a significant effect for Stimulus, due to higher ratings for pain stimuli (*M* = 5.32 ± 1.45) than for no-pain stimuli (*M* = 0.49 ± 0.74), *F*(1, 35) = 634.77, *p* < 0.001. However, a significant interaction effect between Stimulus and Perspective (Figure 1) was also found, *F*(2, 70) = 3.29, *p* < 0.05. Firstly, and according to our hypothesis, the difference in ratings of the pain and no-pain stimuli was calculated for each of the three types of perspectives. This difference was significantly higher for the ingroup perspective (*M* = 5.11 ± 1.25) as compared to the outgroup perspective (*M* = 4.55 ± 1.59), *F*(1, 35) = 5.29, *p* = 0.05. Secondly, we also compared the three types of perspectives with each other for the pain and no-pain stimuli respectively. For the no-pain stimuli, no significant differences were found. In contrast, pain ratings were significantly higher in the ingroup perspective (*M* = 5.54 ± 1.31) than in the self-perspective (*M* = 5.16 ± 1.33) for the pain stimuli, *F*(1, 35) = 6.04, *p* < 0.05, no significant differences being observed with the two others comparisons (self vs. ingroup and ingroup vs. outgroup).

### Response times

The statistical analysis of response times (RTs) revealed a significant main effect for Stimulus, due to longer RTs for pain stimuli (*M* = 2370 ± 847) than for no-pain stimuli (*M* = 1787 ± 961), *F*(1, 35) = 31.11, *p* < 0.001. However, the interaction between Stimulus and Perspective (Figure 1) was significant, *F*(1, 35) = 5.51, *p* < 0.01. Planned comparisons disclosed that the responses for pain stimuli were significantly longer than those for no-pain stimuli in the self-perspective, *F*(1, 35) = 53.80, *p* < 0.001, and in the ingroup perspective, *F*(1, 35) = 18.09, *p* < 0.001.

### Evaluative ingroup bias

The mean evaluation score was positive (*M* = 1.19 ± 1.61) and a one-sample *t*-test revealed that it significantly differs from zero, *t*(1, 35) = 4.45, *p* < 0.001. As shown in Table 1, no correlation coefficients between the subjects’ evaluation scores and the performance in the pain judgment task reached significance.

**Table 1:** **Cognitive (the *Perspective Taking* subscale) and affective (the *Empathic Concern* subscale) subscales of the Interpersonal Reactivity Index (IRI; Davis, 1983; French version: Guttman and Laporte, 2002), evaluation scores, and correlation results**.

	Interpersonal reactivity index				Evaluation score	
	Perspective taking		Empathic concern	
Mean	18.77		19.94		1.19	
SD	4.57		3.86		1.61	
Range	6–27		12–28		−2.4–6.5	

	**Ratings**	**RTs**	**Ratings**	**RTs**	**Ratings**	**RTs**

**PAIN STIMULI**						
Self-perspective	0.05	−0.02	0.23	0.19	0.12	0.04
Ingroup perspective	0.16	−0.08	0.03	0.17	0.14	−0.29
Outgroup perspective	−0.09	−0.16	0.01	0.11	0.03	−0.05
**NO-PAIN STIMULI**						
Self-perspective	−0.02	−0.12	0.04	0.06	0.30	0.25
Ingroup perspective	−0.08	−0.08	−0.39*	−0.19	−0.06	−0.12
Outgroup perspective	0.05	−0.03	−0.29	−0.08	−0.09	0.10
**DIFFERENCE PAIN/NO-PAIN**						
Self-perspective	0.06	0.09	0.23	0.16	0.00	−0.21
Ingroup perspective	0.20	0.01	0.22	0.41*	0.18	−0.17
Outgroup perspective	−0.13	−0.09	0.20	0.19	0.09	−0.15

*p < 0.05.

### Empathy measure

The descriptive scores of the IRI and the correlation results are reported in Table 1. As shown, a significant negative relation between the EC scores and the ratings of no-pain stimuli in the ingroup perspective was observed, *r* = −0.39; *p* < 0.05. Moreover, a positive relation between the EC scores and the RTs for the Pain/No-pain difference in the ingroup perspective reached also significance, *r* = 0.41; *p* < 0.05.

## Discussion

In the present study, we replicated previous findings showing that the mere assignment of individuals to arbitrary groups (i.e., the MGP) elicits evaluative preferences for ingroup relative to outgroup members (Brewer, 1979). More interestingly, we found that the mere act of categorizing people in two distinct social groups is also sufficient to elicit an ingroup bias in empathy for pain^1^. Indeed, participants rated pain stimuli as more painful when they had to adopt the perspective of an ingroup’s member as compared to their own perspective, while the outgroup perspective did not induce different responses to painful pictures as compared to the self-perspective. Moreover, the ratings differences between the painful and non-painful pictures were more important in the ingroup perspective than in the outgroup perspective. Taken together, these observations are consistent with a more general empathic bias toward ingroup members previously shown in the field of intergroup relations (e.g., Brown et al., 2006; Tarrant et al., 2009). For example, some authors found that participants reported stronger empathy when a student in distress belonged to an ingroup compared to an outgroup university (Tarrant et al., 2009). In a similar way, Brown and collaborators (2006) provided evidence for an empathic ingroup bias, participants showing exaggerated affective responses to positive and negative pictures depicting ingroup members.

Contrary to previous studies (e.g., Jackson et al., 2006; Li and Han, 2009), we did not find that taking self-perspective induced faster reactions to perceived pain. In contrast, we found participants to respond more quickly to non-painful than painful stimuli when taking self or ingroup perspective, but not when taking outgroup perspective. Such a discrepancy may be due to methodological differences. In previous researches, response times were collected based on two-alternative forced-choice tasks (i.e., subjects had to judge painful vs. non-painful pictures on each trial) so that the subjects can assign a simple, repeatable, and differentiated motor response to each decision. In contrast, in the present case, reaction times were measured as participants had to rate the level of perceived pain according to the different perspectives. Thus, slower reactions to perceived pain in the self and in group-perspectives could be due to the more care took in accurately assigning a number to pain felt, or alternatively, perhaps an additional stage of processing. Regardless of eventual explanations, it is worth to notice that the same pattern was found when participants had to adopt their own perspective or the one of an ingroup’s member, suggesting the implication of the same mechanisms in these two conditions.

It is well documented in social psychological literature that when two individuals become close, the other is integrated into the self-concept (e.g., Aron et al., 1991). In this vein, Smith and Henry (1996) demonstrated that self-other overlap in mental representations was more important for individuals defined as ingroup members as compared to outgroup members. Thus, an evaluative ingroup bias would be based on the individuals’ proclivity to extend their positive self-representations to encompass their groups (e.g., Cadinu and Rothbart, 1996; Otten and Epstude, 2006). In a similar way, to the extent that similarity between oneself and another may have an important impact on the level of empathy experienced toward another (e.g., Batson et al., 1995; Perry et al., 2010), this overlapping mental representations of the self and the ingroup may also explain the empathic ingroup bias. As compared to previous studies showing that failures of empathy toward outgroup members potentially make them likely targets for prejudice and discrimination (e.g., Gutsell and Inzlicht, 2010), we did not find, however, any relationship between the evaluative and empathic ingroup biases.

Our results also revealed that the cognitive (*PT*) subscale of the (IRI; Davis, 1983; French version: Guttman and Laporte, 2002) did not predict the participants’ performance in the pain judgment task. This suggests that ingroup bias in empathy for pain would not be based on the individuals’ abilities to adopt the perspective of the other. In contrast, the pain judgments in the ingroup perspective were correlated to the EC subscale of the IRI. This confirms the critical role of the affective component, i.e., the affect produced in response to someone else’s emotional state, in empathic ingroup bias for pain, as suggested by antecedent neural investigations (e.g., Xu et al., 2009).

In conclusion, the present study provided new evidence that an observer feels more empathy for someone in pain when that person is in the same social group. But more importantly, we showed for the first time that the mere categorization of individuals on the basis of minimal criteria is sufficient to elicit such an ingroup bias in empathy for pain. Further investigations should be conducted to explore the neural basis of this minimal ingroup bias in line with previous neural researches on empathy for pain in intergroup contexts (Xu et al., 2009; Avenanti et al., 2010; Gutsell and Inzlicht, 2010; for a review, see Chiao and Mathur, 2010).

## Conflict of Interest Statement

The authors declare that the research was conducted in the absence of any commercial or financial relationships that could be construed as a potential conflict of interest.

## Footnotes

^1^One might argue that by highlighting the distinction between the two groups – or introducing information relevant to power or status relations (see Method section), they were not minimal in the accepted sense (Tajfel et al., 1971). Thus, the effects we found would be a function of interpretations of the content we provided – whereas additional information was devoid of any positive or negative evaluation– rather than the mere categorization. It is worth to notice that some authors have used a similar procedure (Ashburn-Nardo et al., 2001; Pinter and Greenwald, 2011), but none of them pointed this aspect. Moreover, given that our method does not allow us to clarify this issue, further investigations should be conducted in order to make sure that the mere act of categorizing people in two distinct social groups is sufficient to elicit an ingroup bias in empathy for pain, for example by comparing performances in two paradigms with and without these instructions.
